# CDX2 inhibits the proliferation and tumor formation of colon cancer cells by suppressing Wnt/β-catenin signaling via transactivation of GSK-3β and Axin2 expression

**DOI:** 10.1038/s41419-018-1263-9

**Published:** 2019-01-10

**Authors:** Junhui Yu, Dong Liu, Xuejun Sun, Kui Yang, Jianfeng Yao, Chen Cheng, Chunbao Wang, Jianbao Zheng

**Affiliations:** 1 Department of General Surgery, First Affiliated Hospital of Xi’an Jiaotong University, Xi’an, 710061 Shaanxi Province China; 2 Second Department of General Surgery, Shaanxi Provincial People’s Hospital, Xi’an, 710068 Shaanxi Province China; 3 First Department of General Surgery, Shaanxi Provincial People’s Hospital, Xi’an, 710068 Shaanxi Province China; 4 Department of Pathology, First Affiliated Hospital of Xi’an Jiaotong University, Xi’an, 710061 Shaanxi Province China

## Abstract

Caudal-related homeobox transcription factor 2 (CDX2), an intestine-specific nuclear transcription factor, has been strongly implicated in the tumourigenesis of various human cancers. However, the functional role of CDX2 in the development and progression of colorectal cancer (CRC) is not well known. In this study, CDX2 knockdown in colon cancer cells promoted cell proliferation in vitro, accelerated tumor formation in vivo, and induced a cell cycle transition from G0/G1 to S phase, whereas CDX2 overexpression inhibited cell proliferation. TOP/FOP-Flash reporter assay showed that CDX2 knockdown or CDX2 overexpression significantly increased or decreased Wnt signaling activity. Western blot assay showed that downstream targets of Wnt signaling, including β-catenin, cyclin D1 and c-myc, were up-regulated or down-regulated in CDX2-knockdown or CDX2-overexpressing colon cancer cells. In addition, suppression of Wnt signaling by XAV-939 led to a marked suppression of the cell proliferation enhanced by CDX2 knockdown, whereas activation of this signaling by CHIR-99021 significantly enhanced the cell proliferation inhibited by CDX2 overexpression. Dual-luciferase reporter and quantitative chromatin immunoprecipitation (qChIP) assays further confirmed that CDX2 transcriptionally activates glycogen synthase kinase-3β (GSK-3β) and axis inhibition protein 2 (Axin2) expression by directly binding to the promoter of GSK-3β and the upstream enhancer of Axin2. In conclusion, these results indicated that CDX2 inhibits the proliferation and tumor formation of colon cancer cells by suppressing Wnt/β-catenin signaling.

## Introduction

Globally, colorectal cancer (CRC) is the third most common cancer and ranks as the fourth leading cause of cancer death^1^. Although the multimodality therapy for CRC has achieved great progress, most advanced CRC patients have a poor prognosis. The 5-year survival rate of patients with stage I CRC is >90%; however, the rate of patients with stage IV CRC is slightly >10%^2^. An increasing number of genetic and molecular alterations have been recognized in colorectal carcinogenesis, including genetic mutations, microsatellite instability, and DNA hypermethylation^3,4^. Thus, elucidating the molecular mechanisms of CRC pathogenesis is critical for providing a better strategy for treating CRC^5^.

Canonical Wnt signaling performs a crucial role in maintaining intestinal homeostasis by regulating proliferation, differentiation, and cell-fate decisions^6–8^. Aberrant activation of Wnt signaling is associated with human carcinogenesis, including CRC^9,10^. Mutations or dysregulation of the β-catenin destruction complex (APC, Axin2, CK1, and GSK-3β) results in activation of Wnt signaling^11–13^. Furthermore, an elevated nuclear β-catenin level is considered a hallmark of invasive CRC, leading to the activation of Wnt-related targets, including c-myc, cyclin D1, MMP2, and MMP9, thereby promoting cell proliferative, invasive, and migratory potential^14–17^.

Caudal-related homeobox transcription factor 2 (CDX2), an intestine-specific nuclear transcription factor, regulates the balance between cell proliferation and differentiation in intestinal epithelium^18^. Activation of CDX2 affects the cytodifferentiation and villus morphology of murine intestinal epithelial cells^19^. Recently, increasing evidence supports a potential role of CDX2 as an oncogene or suppressor in tumourigenesis of various human malignancies including hepatocellular carcinoma^20^, pancreatic cancer^21,22^, lung cancer^23,24^, and gastric cancer^25,26^_._ In human CRC, a CDX2 reduction is inversely related to tumor grade, lymph node metastasis, tumor stage, and a poor prognosis^27,28^. Our previous study indicated that restoration of CDX2 expression markedly suppressed the aggressive phenotype of colon cancer cells, including viability, colony formation, and invasive and migratory abilities^29–31^. Furthermore, CDX2^+/−^ mice were susceptible to developing colon tumor^32^. Recent evidence indicated that in lung cancer, overexpression of CDX2 inhibits β-catenin/TCF activity and consequential downstream molecular^24^. However, the role of CDX2 in regulating Wnt signaling in human CRC development and progression remain to be elucidated.

In this study, we aim to investigate the correlation between CDX2 expression and its target genes involved in Wnt/β-catenin signaling during tumourigenesis in human CRC.

## Materials and methods

### Clinical samples and cell cultures

Twenty human CRC tissues were obtained from patient diagnosed with CRC and received surgery at the First Affiliated Hospital of Xi’an Jiaotong University from January 2016 to September 2016. No patient had received preoperative chemotherapy or radiotherapy. Informed consents were signed by all patients, and the study protocol was approved by the Ethics Committee of the First Affiliated Hospital of Xi’an Jiaotong University.

HT-29 and Caco-2 cells (Shanghai Institute of Cell Biology, Chinese Academy of Sciences) were maintained in RPMI-1640 medium (Gibco BRL, Carlsbad, CA, USA) supplemented with 10% FBS (Gibco BRL, Carlsbad, CA, USA) at 5% CO_2_ at 37 °C.

### Lentiviral vectors and transfection

The phU6-EGFP-shRNA-CDX2 lentiviral vectors and their control vectors were used to inhibit CDX2 expression, while the pUbi-EGFP-CDX2 lentiviral vectors and their control vectors were used to increase CDX2 expression. All the lentiviral vectors constructed and prepared by GeneChem Co., Ltd. (Shanghai, China). The target shRNA sequence was 5′-ACAAATATCGAGTGGTGTA-3′. All transfections were performed according to the manufacturer’s instructions.

### Cell growth and cell viability assays

HT-29 and Caco-2 cells were transfected with lentiviral vectors as described above. For cell growth, cells were seeded into 35-mm culture dishes for 7 days. The cells were counted using a haemocytometer under a light microscope every 2 days. For cell viability assays, cells were seeded into 96-well culture plates at 3000 cells/well for 4 days. Cell viability was examined using the CCK-8 assay (Dojindo, Tokyo, Japan) every 2 days by following the manufacturer’s protocol.

### Cell cycle assay

Cells were harvested and fixed in 75% cold ethanol and stored at 4 °C overnight. After treatment with RNase A at 37 °C for 30 min, the cells were incubated with propidium iodide in the dark for 30 min. The cell cycle was assessed with flow cytometry (BD, Franklin Lakes, NJ, USA).

### Nude mouse xenograft assay

The use of all animals in this study was approved by the Institutional Animal Care and Use Committee of the First Affiliated Hospital of Xi’an Jiaotong University. Tumor cells (5 × 10^6^) in logarithmic phase were subcutaneously injected into the right flanks of 5-week-old female BALB/c-nude mice (Shanghai SLAC Laboratory Animal Co. Ltd., Shanghai, China). After 6 days post-injection, the length (*a*) and width (*b*) of the tumor were monitored using callipers every 3 days. The tumor volume (*V*) was calculated as follows: *V* = *ab*^2^/2. At the end of the experiment, the mice were sacrificed and the xenograft tumors were measured.

### RNA isolation and real-time PCR

Total RNA was isolated from cells using TRIzol reagent (Invitrogen, Carlsbad, CA, USA). Complementary DNA (cDNA) was synthesized by the PrimeScript RT Reagent Kit (TaKaRa, Osaka, Japan). Real-time PCR was conducted on an IQ5 instrument (Bio-Rad, CA, USA) using SYBR Green fluorescence signal detection assays (TaKaRa, Osaka, Japan) with primers (Table 1). The specific mRNA expression level was quantified by using the 2^−∆∆CT^ method.

**Table 1:** Primer sequence

Gene	Sequence
APC for qRT-PCR	F: 5′-AAAATGTCCCTCCGTTCTTATGG-3′
	R: 5′-CTGAAGTTGAGCGTAATACCAGT-3′
Axin2 for qRT-PCR	F: 5′-AGCCAAAGCGATCTACAAAAGG-3′
	R:5′-AAGTCAAAAACATCTGGTAGGCA-3′
GSK-3β for qRT-PCR	F: 5′-GGCAGCATGAAAGTTAGCAGA-3′
	R: 5′-GGCGACCAGTTCTCCTGAATC-3′
GAPDH for qRT-PCR	F: 5′-TGCACCACCAACTGCTTAGC-3′
	R: 5′-GGCATGGACTGTGGTCATGAG-3′
GSK-3β promoter for PCR	F: 5′-GGGGTACCTGTTAAATATCCGT GCCGATCT-3′
	R: 5′-CCAAGCTTAGGAGGTCTAATAA TTTCAGATCCT-3′
Axin2 promoter for PCR	F: 5′-GGGGTACCGCTGGAAACAGGACC TGCGT-3′ R: 5′- CCAAGCTTGACAGGCATGGGTTT GGTGA-3′
Axin2 for CHIP-qPCR	5′-GGAGCAGTAAAAGGCCGTAA-3′
	5′-CCAAACCATTGAAGCCCTTA -3′
GSK-3β for CHIP-qPCR	5′-CAGAGACGCTGGTGAAACTG-3′
	5′-CCCCTTCTCTTCACCAATCA-3′

### Immunohistochemistry (IHC)

For IHC, the staining procedure was performed using the standard avidin–biotin complex method as previously described^33^. The CDX2-stained, β-catenin, c-myc-stained, cyclin D1-stained sections were divided into two groups (negative and positive) based on the extent and intensity of the staining. The extent of positively stained cells was scored on a scale from 0 to 4: 0–5% (0), 6–25% (1), 26–50% (2), 51–75% (3), and 76–100% (4). The staining intensity was scored on a scale from 0 to 3: negative (0), weakly positive (1), moderately positive (2), and strongly positive (3). The immunoreactivity score (IRS) is defined as the product of the extent score and the intensity score. An IRS of ≤3 was defined as negative, and a score of >3 was defined as positive. Two pathologists evaluated all the specimens in a blinded manner.

### Protein extraction and Western blotting

Cells were lysed using RIPA buffer (Heart, Xian, China). Cell lysates containing 50 μg of total protein were then subjected to SDS–PAGE (Beyotime, Shanghai, China) and then transferred to PVDF membranes (Millipore, Billerica, MA, USA). The membranes were incubated with primary antibodies overnight at 4 °C (anti-CDX2, Axin2, APC, GSK-3β, β-catenin, cyclin D1, c-myc, or GAPDH, 1:1000 dilution). The membrane was then washed four times with TBST buffer for 8 min each and incubated with a horseradish peroxidase-conjugated secondary antibody at room temperature for 1 h. Chemiluminescent HRP substrate (Millipore, Billerica, MA, USA) was added to visualize the protein bands. The antibodies against c-myc, β-catenin, and GAPDH were purchased from Santa Cruz (Dallas, TX, USA), the antibodies against Axin2 and APC were purchased from Abcam (Cambridge, MA, USA), and the antibodies against CDX2 and GSK-3β were purchased from Cell Signaling Technology (Danvers, MA, USA).

### Luciferase reporter assay

Fragments of the Axin2 (from position –2508 to +153 bp) and the GSK-3β 5′-flanking sequence (from position –222 to +180 bp) were amplified PCR using primers (Table 1) using primers (Table 1) and cloned into the luciferase reporter vector pGL3.0-Basic (Promega, Madison, WI, USA) to generate Axin2 and GSK-3β promoter reporter constructs. The AXIN2 enhancer region (position −15,083 to −16,104 bp) was cloned into Axin2 promoter reporter constructs. Plasmids containing firefly luciferase reporters and pTK-RL plasmids were co-transfected into cells. For the TOP/FOP-Flash reporter assay, the TOP/FOP-Flash reporter and pTK-RL plasmids were co-transfected into cells. After transfection for 48 h, the cells were harvested for analysis with the Dual-Luciferase Reporter Assay System (Promega, Madison, WI, USA). Luciferase activity was measured using the PerkinElmer EnSpire Multilabel Reader 2300 (PerkinElmer Inc., Waltham, MA, USA). The luciferase intensity was normalized to the Renilla luciferase activity to normalize for transfection efficiency.

### Quantitative chromatin immunoprecipitation (qChIP)

Cells were subjected to ChIP using the EZ-ChIP Kit (Millipore, Bedford, MA, USA) according to the manufacturer’s protocol. The chromatin–protein complexes were incubated with anti-CDX2 antibodies or rabbit IgG. Real-time PCR was conducted to amplify the regions of interest by using primers (Table 1).

### Statistical analysis

Each experiment was repeated three times. Data are presented as the mean ± SD. The chi-square test or one-way ANOVA was performed to compare the differences among the groups. Correlations were analyzed using Pearson linear-regression analysis. Statistical analyses were performed with SPSS 18.0 software (SPSS Inc., Chicago, IL, USA). *P* < 0.05 was considered statistically significant.

## Results

### CDX2 inhibits tumor formation of colon cancer cells in vivo

HT-29 and Caco-2 cells were transfected with CDX2-shRNA or CDX2-overexpressing lentivirus to generate stable CDX2-knockdown or CDX2-overexpressing cells (Supplementary Fig. 1a–d, all *P* < 0.05).

**Fig. 1:**
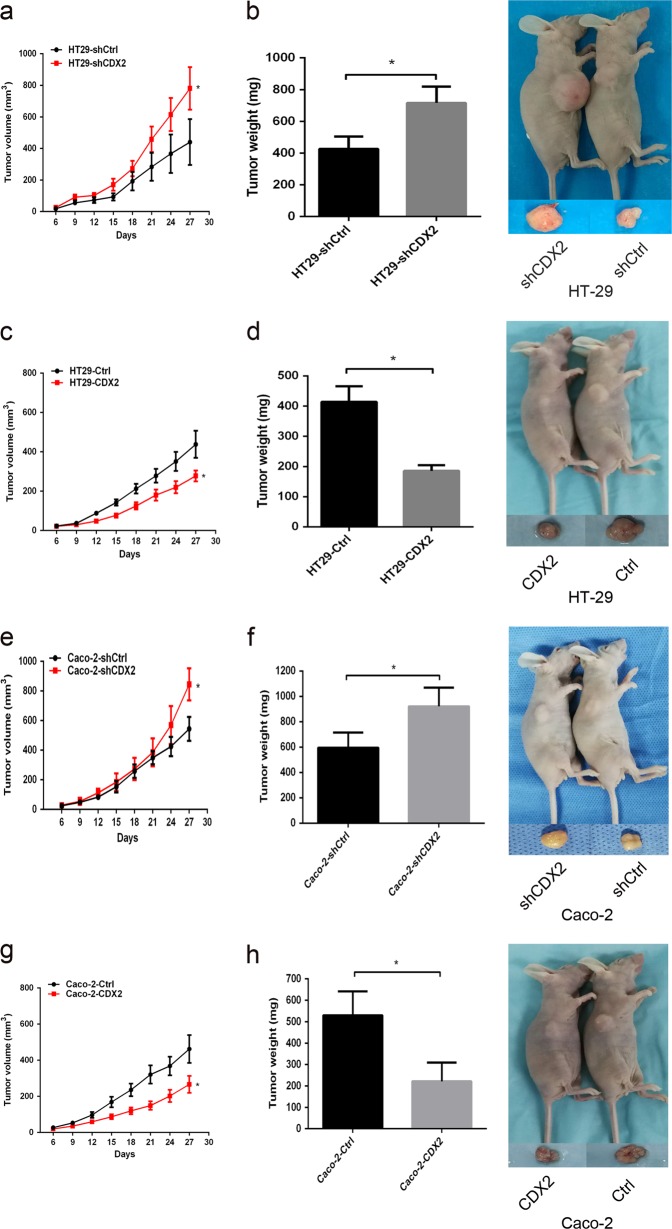
CDX2 inhibits the tumor formation of colon cancer cells in vivo. a, b. Tumor growth curves (**a**) and tumor weights (**b**) were shown for CDX2-knockdown HT-29 cells. **c**, **d** Tumor growth curves (**c**) and tumor weights (**d**) were shown for CDX2-overexpressing HT-29 cells. e, f Tumor growth curves (**e**) and tumor weights (**f**) were shown for CDX2-knockdown Caco-2 cells. **g**, **h** Tumor growth curves (**g**) and tumor weights (**h**) were shown for CDX2-overexpressing Caco-2 cells. *P < 0.05

To evaluate the role of CDX2 in the tumor formation ability in vivo, the CDX2-modified colon cancer cells and their control cells were injected subcutaneously into nude mice. The xenograft tumors in CDX2-knockdown group were larger and heavier than those in the control group (Fig. 1a, b, e, f, all P < 0.05). Conversely, the xenograft tumors in CDX2-overexpressing group developed much more slowly and were lighter than those in the control group (Fig. 1c, d, g, h, all *P* < 0.05).

**Fig. 2:**
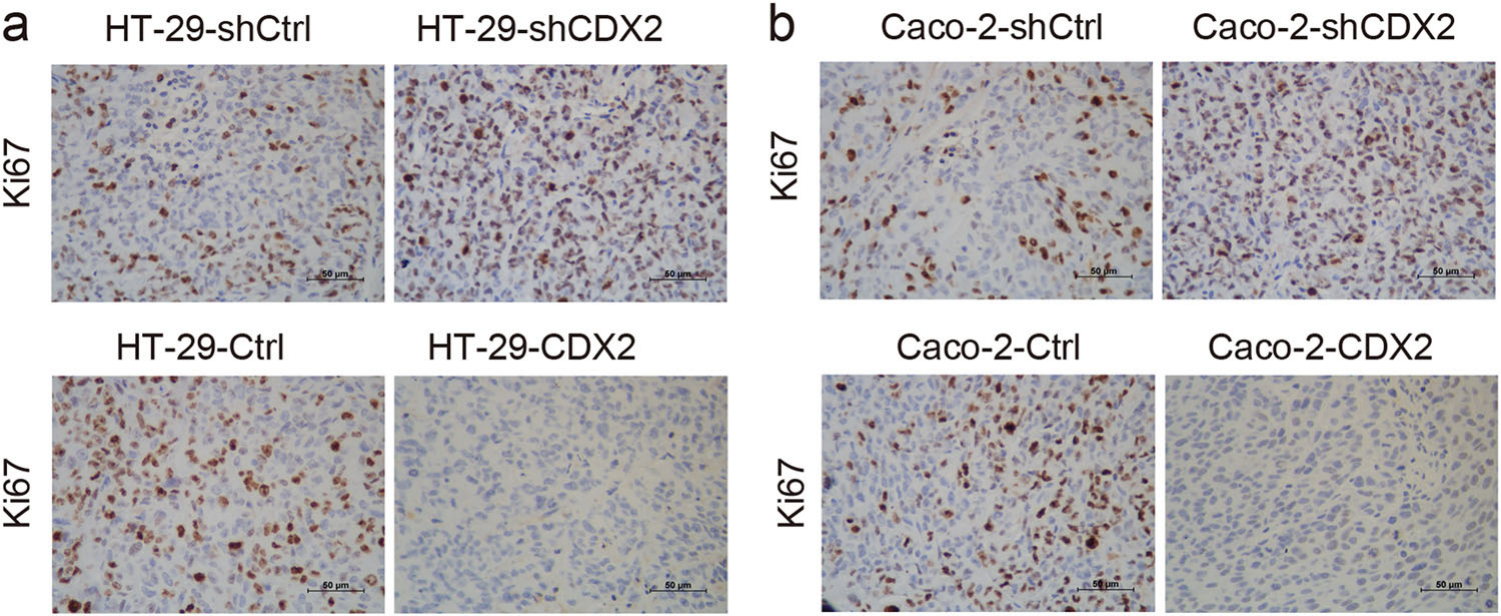
Immunohistochemical staining for Ki67 is shown in tumor xenografts of CDX2 knockdown and overexpressing HT-29 (**a**) and Caco-2 (**b**) cells

To investigate whether the tumor suppression potential of CDX2 in vivo could be due to its cell proliferation inhibition, the expression levels of the universal proliferation biomarker Ki67 were determined by IHC. The results showed the xenograft tumor tissues formed by CDX2-knockdown cells demonstrated a much stronger Ki67-staining score than those formed by the control cells (Fig. 2a, b), whereas the xenograft tumor tissues formed by CDX2-overexpressing cells had the opposite effect (Fig. 2a, b). These results suggested that CDX2 suppressed the tumor formation of colon cancer cells, potentially by inhibiting cell proliferation in vivo.

### CDX2 inhibits the proliferation of colon cancer cells by arresting the transition from G0/G1 to S phase

**Fig. 3:**
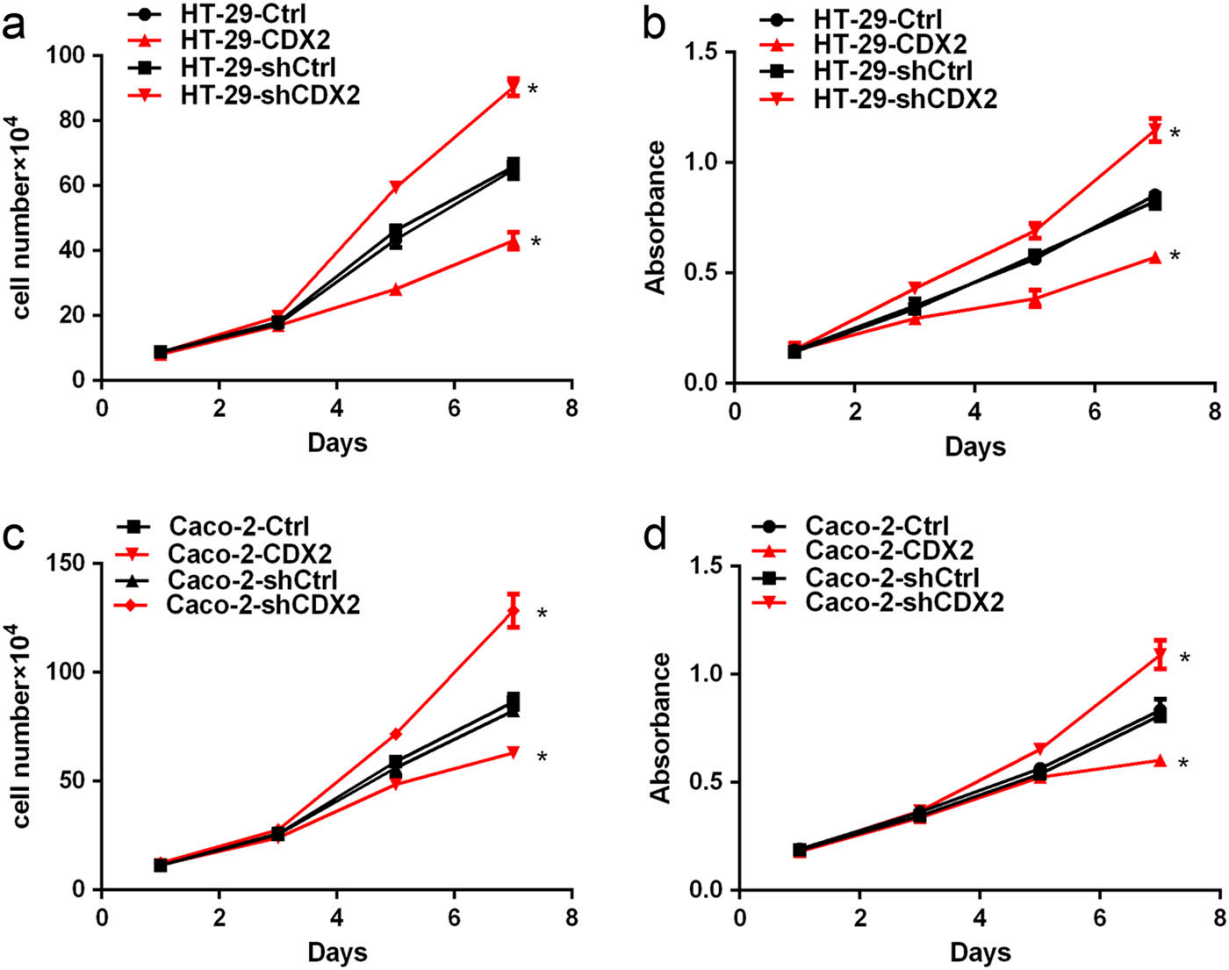
CDX2 inhibits the proliferation of colon cancer cells. **a**, **b** Cell growth and MTT assays were performed using CDX2-knockdown (**a**) and CDX2-overexpressing (**b**) HT-29 cells. **c**, **d** Cell growth and MTT assays were performed using CDX2-knockdown (**c**) and CDX2-overexpressing (**d**) Caco-2 cells. All data are the mean ± SD of three independent experiments. *P < 0.05

We next performed a cell growth curve assay and an MTT assay to evaluate the tumor growth inhibition of CDX2 in vitro. Our study showed that CDX2 knockdown led to significant promotion of the cell growth and viability (Fig. 3a–d, all *P* < 0.05). Conversely, overexpression of CDX2 significantly inhibited cell growth and viability (Fig. 3a–d, all *P* < 0.05). These data suggested that CDX2 could inhibit colon cancer cell proliferation in vitro.

**Fig. 4:**
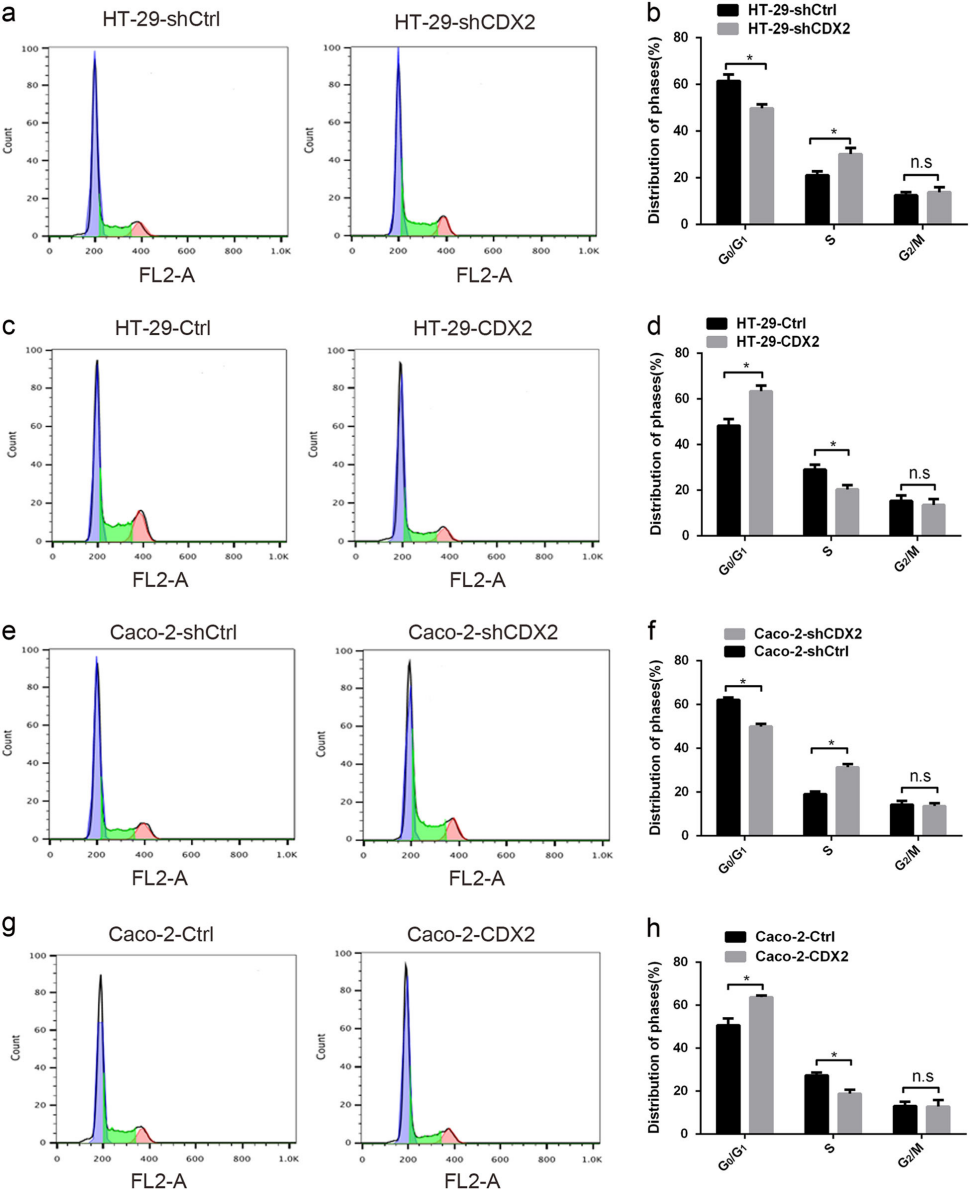
CDX2 inhibits the proliferation of colon cancer cells by arresting the cell cycle transition from G0/G1 to S phase. **a** Fluorescence-activated cell sorting (FACS) analysis of the cell cycle distribution was shown for CDX2-knockdown HT-29 cells. **b** Comparison of the cell cycle distribution of CDX2-knockdown HT-29 cells. **c** The FACS analysis of cell cycle distribution was shown for CDX2-overexpressing HT-29 cells. **d** Comparison of the cell cycle distribution of CDX2-overexpressing HT-29 cells. **e** The FACS analysis of the cell cycle distribution was shown for CDX2-knockdown Caco-2 cells. **f** Comparison of the cell cycle distribution of CDX2-knockdown Caco-2 cells. **g** The FACS analysis of the cell cycle distribution was shown for CDX2-overexpressing Caco-2 cells. **h** Comparison of the cell cycle distribution of CDX2-overexpressing Caco-2 cells. All data are the mean ± SD of three independent experiments. *P < 0.05

To reveal the potential mechanism of the CDX2-mediated inhibition of colon cancer cell proliferation, flow cytometry was conducted to evaluate the cell cycle of CDX2-modified HT-29 and Caco-2 cells. CDX2 knockdown in colon cancer cells led to a decreased percentage of cells in the G0/G1 phase and an increased percentage in the S phase compared to the control (Fig. 4a, b, e, f, all *P* < 0.05). Conversely, CDX2 overexpression in colon cancer cells decreased the proportion of cells in the S phase and increased the proportion in the G0/G1 phase (Fig. 4c, d, g, h, all *P* < 0.05). No significant change was found in the percentage of cells in the G2 phase in CDX2-knockdown or CDX2-overexpressing cells (all *P* > 0.05). Our study demonstrated that CDX2 inhibits colon cancer cell proliferation by delaying the transition from G0/G1 to S phase.

### CDX2 attenuates the Wnt signaling activity in colorectal carcinogenesis

**Fig. 5:**
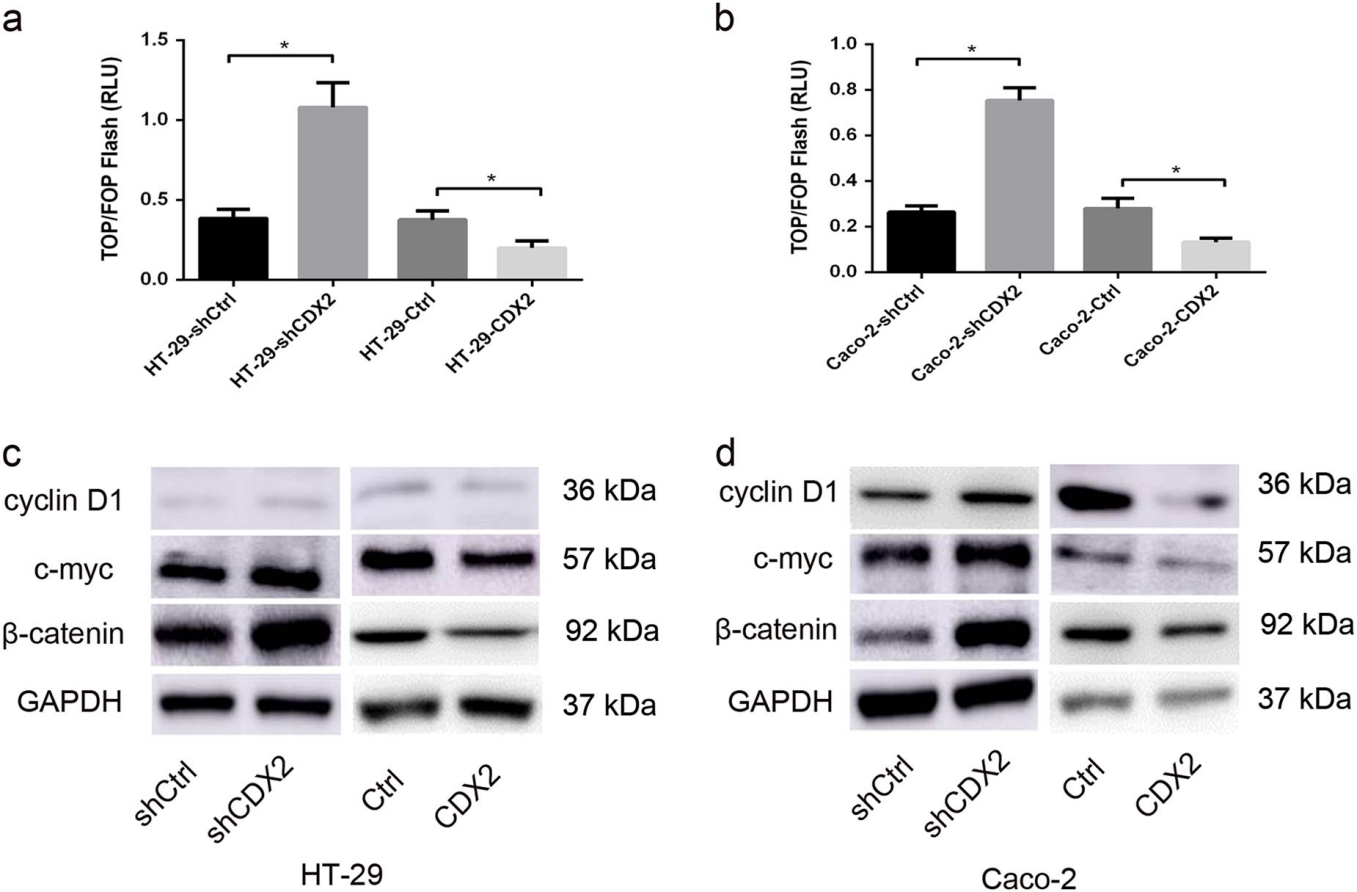
CDX2 attenuates the activity of the Wnt/β-catenin pathway in colorectal carcinogenesis. **a**, **b** The luciferase reporter activities in CDX2-knockdown and CDX2-overexpressing HT-29 (**a**) or Caco-2 (**b**) cells transfected with a TOP/FOP-Flash reporter plasmid. **c**, **d** Western blot bands of cyclin D1, c-myc, and β-catenin in CDX2-knockdown and CDX2-overexpressing HT-29 (**c**) and Caco-2 (**d**) cells. All data are the mean ± SD of three independent experiments. *P < 0.05

Recent studies indicated that CDX2 acts as a negative regulator of Wnt/β-catenin signaling in lung cancer^24^ and CRC^34^. However, to the best of our knowledge, the precise mechanisms of the CDX2-mediated inhibition of colon cancer cell proliferation have not been proved to be associated with its suppression of Wnt signaling. Therefore, the TOP/FOP-Flash reporter assay was performed to evaluate the Wnt signaling activity in CDX2-modified cells^35^. The luciferase activities were higher in CDX2-knockdown cells, compared to the control cells (Fig. 5a, b, all *P* < 0.05). Conversely, the luciferase activities were lower in CDX2-overexpressing cells (all *P* < 0.05). These findings indicate that CDX2 inhibits the Wnt/β-catenin signaling activity in colon cancer cells.

Cyclin D1, c-myc, and β-catenin are well-known Wnt-related genes that regulate the cell cycle. In accordance with the increased activity of Wnt signaling, the expression levels of cyclin D1, c-myc, and β-catenin in CDX2-knockdown cells were all increased compared to that in the control cells (Fig. 5c, d, supplementary Fig. 2a and c, all *P* < 0.05). However, in CDX2-overexpressing cells, the expression levels of the cyclin D1, c-myc, and β-catenin proteins were decreased (Fig. 5c, d, supplementary Fig. 2b and d, all *P* < 0.05). These findings confirm that CDX2 functions as a negative mediator of Wnt signaling in colon cancer cells.

### Suppression of Wnt signaling by XAV-939 abolishes cell proliferation enhanced by CDX2 knockdown in colon cancer cells

To further support that Wnt signaling is involved in the mechanism by which CDX2 inhibits cell proliferation in human CRC, XAV-939, an inhibitor of Wnt signaling that acts by stabilizing Axin2, was administered to suppress the Wnt signaling in CDX2-knockdown cells^36^. Treatment with XAV-939 decreased the protein levels of cyclin D1, c-myc, and β-catenin in CDX2-knockdown cells (Supplementary Fig. 3a–d, all *P* < 0.05). Consistent with the down-regulation of these Wnt-related proteins, treatment with XAV-939 led to significant abolishment of the cell growth and viability of CDX2-knockdown cells (Supplementary Fig. 3e–h, all *P* < 0.05).

Furthermore, CDX2-overexpressing cells were treated with CHIR-99021, which suppresses β-catenin degradation by inhibiting GSK-3β^37^, to activate Wnt/β-catenin signaling. Our study showed that treatment with CHIR-99021 restored the protein levels of cyclin D1, c-myc, and β-catenin inhibited by overexpression of CDX2 (Supplementary Fig. 4a–d, all *P* < 0.05), as well as the cell growth and viability (Supplementary Fig. 4e–h, all *P* < 0.05). These findings further confirm that CDX2-mediated cell proliferation inhibition in human CRC might be strongly related to its suppression of Wnt signaling.

### CDX2 attenuates Wnt/β-catenin signaling by directly transactivating GSK-3β and Axin2 expression in colon cancer cells

**Fig. 6:**
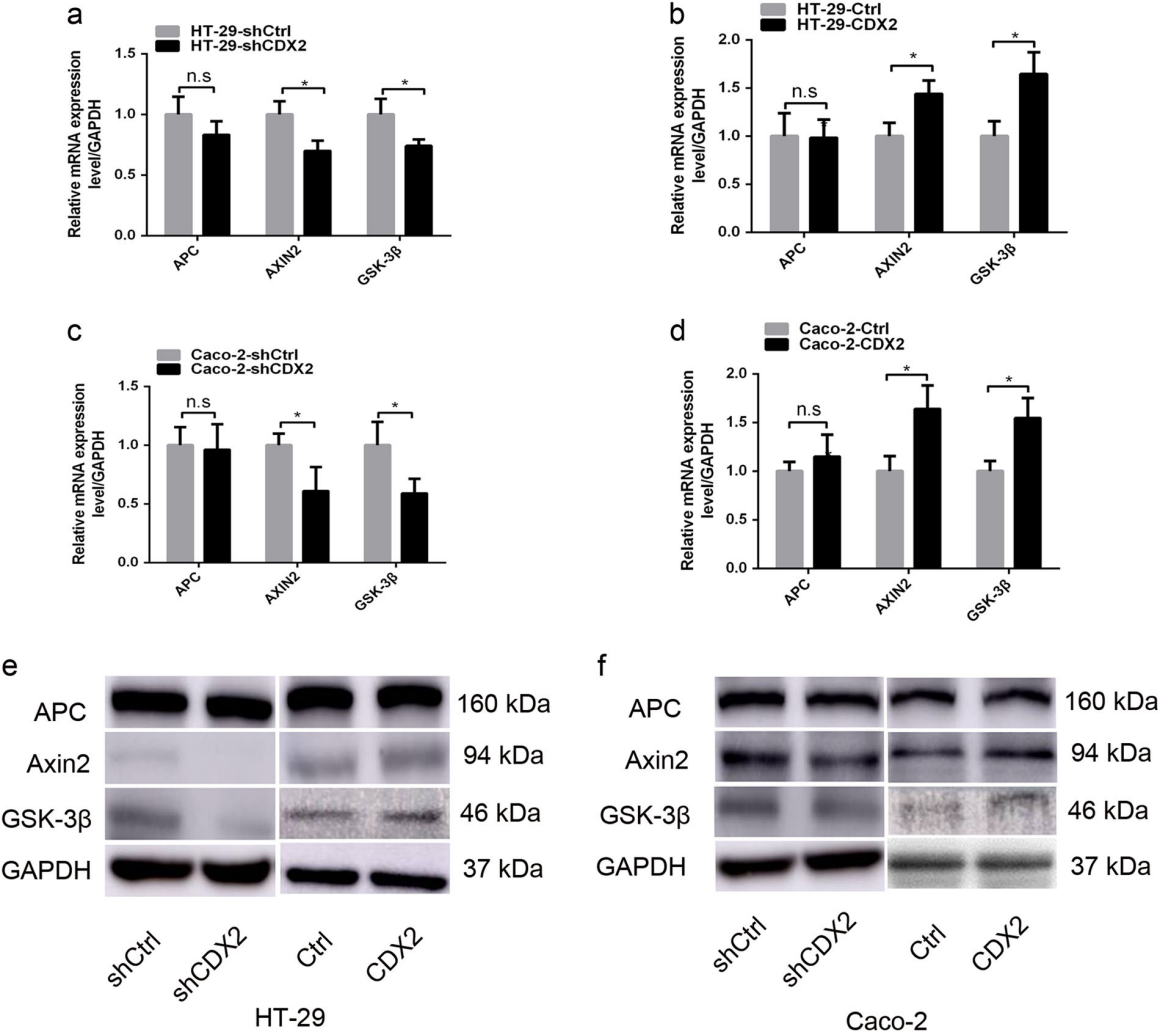
CDX2 regulates the expression of GSK-3β and Axin2 in colon cancer cells. **a**, **b** The mRNA expression of APC, Axin2, and GSK-3β in CDX2-knockdown (**a**) and CDX2-overexpressing (**b**) HT-29 cells. **c**, **d** The mRNA expression of APC, Axin2, and GSK-3β in CDX2-knockdown (**c**) and CDX2-overexpressing (**d**) Caco-2 cells. e, f Western blot bands of APC, Axin2, and GSK-3β in CDX2-knockdown and CDX2-overexpressing HT-29 (**e**) and Caco-2 (**f**) cells. All data are the mean ± SD of three independent experiments. *P < 0.05

The precise molecular mechanisms by which CDX2 suppresses the Wnt signaling activity in colon cancer cells were further elucidated. CDX2 has been reported to bind to the upstream enhancer of APC and AXIN2 and the promoter of GSK-3β^38^. The down-regulation of those proteins would promote β-catenin nuclear translocation and activate Wnt signaling^39^. Our study aimed to determine whether CDX2 suppressed Wnt signaling in colon cancer cells by regulating these genes. Our study found that CDX2 knockdown led to a decrease in the expression levels of GSK-3β and Axin2 compared to those in the control, as determined by real-time PCR (Fig. 6a, c, all *P* < 0.05) and Western blot analysis (Fig. 6e, f, supplementary Fig. 5a and c, all *P* < 0.05). Conversely, CDX2 overexpression resulted in an increase in the expression levels of GSK-3β and Axin2 (Fig. 6b, d, e, f, supplementary Fig. 5b and d, all *P* < 0.05). However, we did not observe a significant change in APC expression in CDX2-modified cells (all *P* > 0.05). These findings implied that CDX2 might suppress Wnt signaling activity by up-regulating GSK-3β and Axin2 expression.

**Fig. 7:**
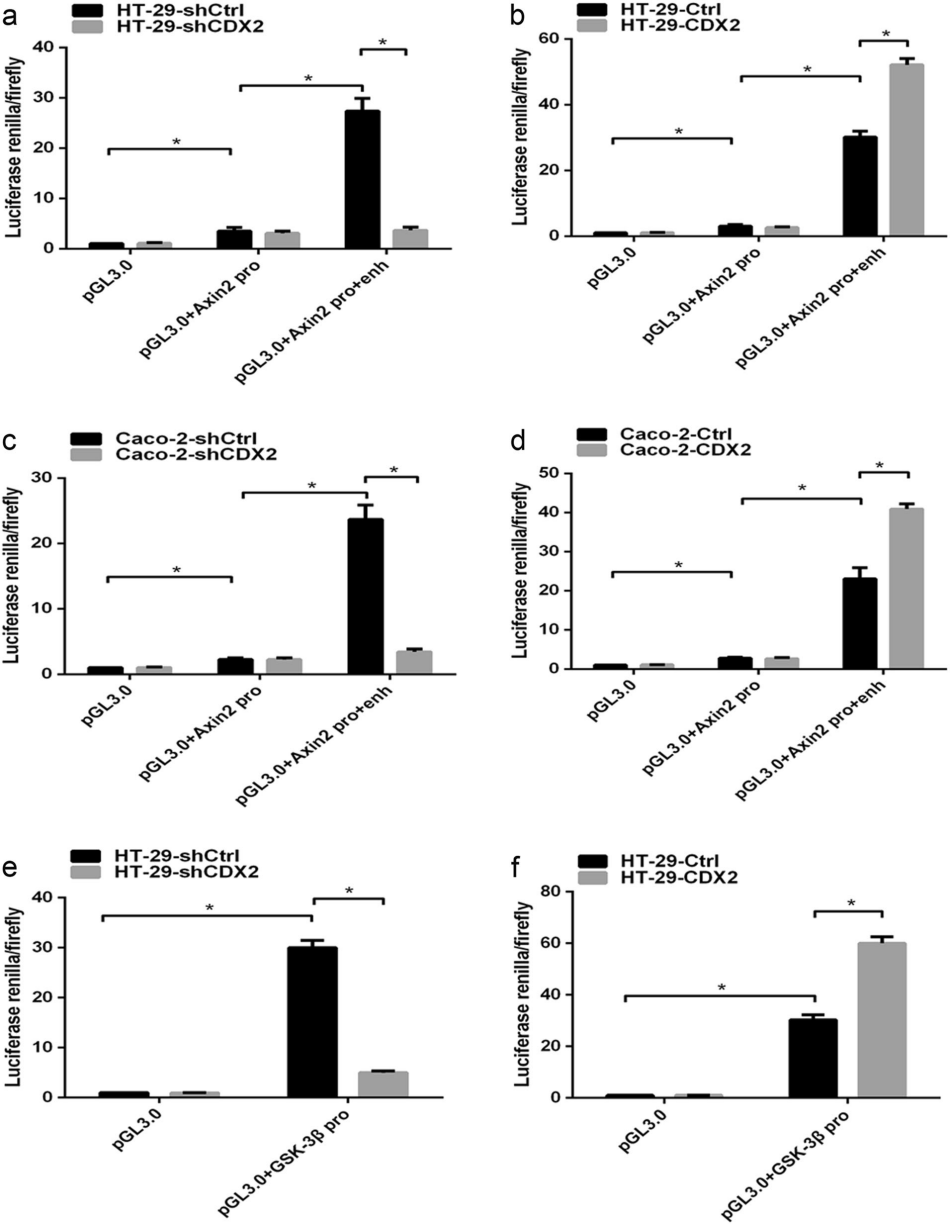

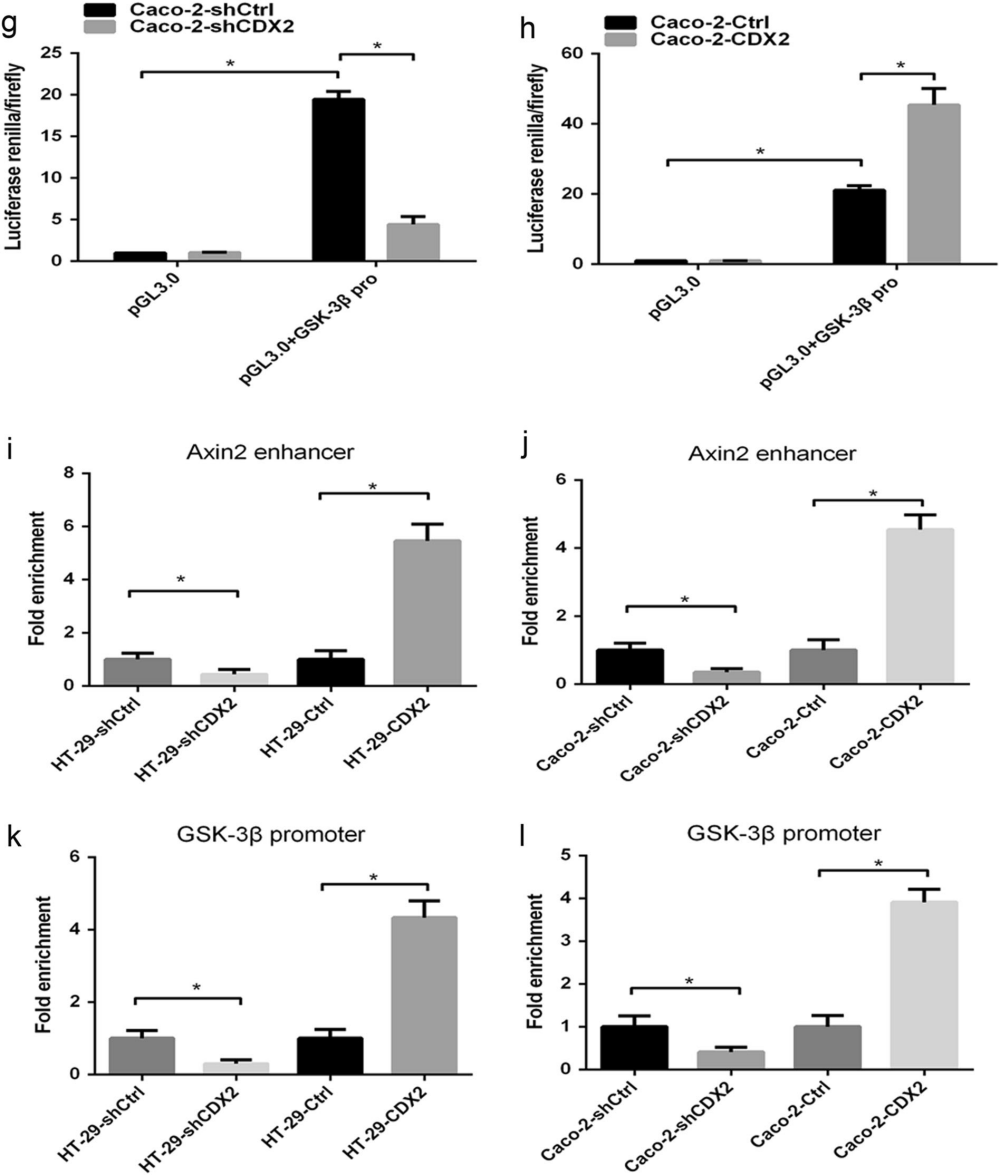
CDX2 attenuates the Wnt/β-catenin pathway through direct transcriptional activation of the expression of GSK-3β and Axin2 in colon cancer cells. **a**, **b** Axin2 promoter activities in CDX2-knockdown (**a**) and CDX2-overexpressing (**b**) HT-29 cells. **c**, **d** Axin2 promoter activities in CDX2-knockdown (**c**) and CDX2-overexpressing (**d**) Caco-2 cells. **e**, **f** GSK-3β promoter activities in CDX2-knockdown (**e**) and CDX2-overexpressing (**f**) HT-29 cells. **g**, **h** GSK-3β promoter activities in CDX2-knockdown (**g**) and CDX2-overexpressing (**h**) Caco-2 cells. **i**, **j** Enrichment level of CDX2 binding to specific regions of the Axin2 enhancer in CDX2-knockdown and CDX2-overexpressing HT-29 (**i**) and Caco-2 cells (**j**) determined by the qChIP assay. **k**, **l** Enrichment level of CDX2 binding to specific regions of the GSK-3β promoter in CDX2-knockdown and CDX2-overexpressing HT-29 (**k**) and Caco-2 cells (**l**) determined by the qChIP assay. All data are the mean ± SD of three independent experiments. *P < 0.05

CDX2, as a transcription factor, regulates the expression of target genes by transactivating them. To confirm the hypothesis that CDX2 transcriptionally activated GSK-3β and Axin2, a dual-luciferase reporter assay was performed. Our study showed that the luciferase activity was slightly higher in HT-29 and Caco-2 cells transfected with the construct containing the Axin2 promoter than in those transfected with the pGL3.0 empty vector (Fig. 7a–d, all *P* < 0.05). However, the transfection of the construct containing the Axin2 promoter and enhancer led to a marked increase in the activity compared to that for the construct containing the Axin2 promoter only (all *P* < 0.05). Furthermore, CDX2 knockdown or CDX2 overexpression attenuated or enhanced the luciferase activity of the construct containing the Axin2 promoter and enhancer (all *P* < 0.05). The luciferase activity increased in HT-29 and Caco-2 cells transfected with the GSK-3β promoter construct compared to that in cells transfected with the empty vector (Fig. 7e–h, all *P* < 0.05). CDX2 knockdown or CDX2 overexpression led to a decrease or increase, respectively, in the luciferase activity of GSK-3β compared to that in the control (all *P* < 0.05).

Furthermore, a quantitative ChIP (qChIP) assay was used to demonstrate that CDX2 directly binds to the GSK-3β promoter and Axin2 enhancer in vivo. Our study revealed an enhancement in CDX2 binding to the GSK-3β promoter and Axin2 enhancer in CDX2-knockdown cells compared to that in the control cells (Fig. 7i–l, all *P* < 0.05). Conversely, the binding of CDX2 to the GSK-3β promoter and Axin2 enhancer was suppressed in CDX2-overexpressing cells (all *P* < 0.05). These findings indicated that CDX2 transactivates GSK-3β and Axin2 expression by directly binding to the GSK-3β promoter and the Axin2 upstream enhancer in colon cancer cells.

In summary, these above results indicated that CDX2 attenuates the Wnt/β-catenin signaling in colon cancer cells by directly transactivating GSK-3β and Axin2 expression.

### Correlation between CDX2 expression and Wnt signaling in human CRC specimens

**Fig. 8:**
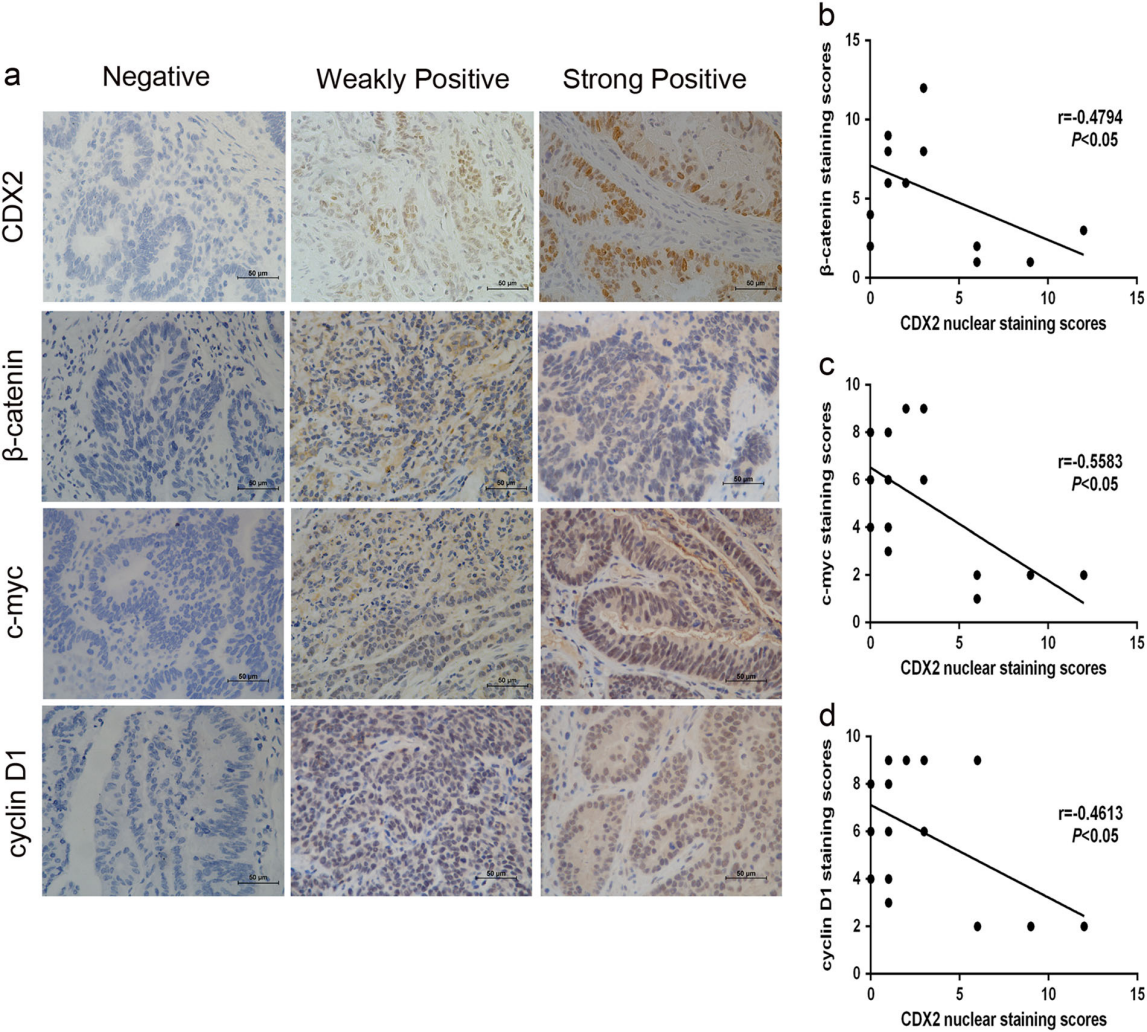
Correlation analysis between the expression of CDX2 and Wnt signaling in human CRC specimens. **a** Immunohistochemical staining showing the CDX2, β-catenin, c-myc, and cyclin D1 expression in CRC tissues. **b**, **c**, **d** Correlation of the CDX2 staining and the β-catenin (**b**, *r* = −0.4794; *P* < 0.05), cyclin D1 (**c**, *r* = −0.5583; *P* < 0.05) and c-myc (**d**, *r* = −4613; *P* < 0.05) staining. *P < 0.05

Immunohistochemical staining was conducted to illustrate the clinical correlation between CDX2 expression and Wnt signaling in human CRC (Fig. 8a). Our study found that CDX2 expression was inversely related to cyclin D1, c-myc, and β-catenin expression in CRC specimens (Fig. 8b–d, all *P* < 0.05), supporting the notion that CDX2 suppressed Wnt signaling in human CRC.

## Discussion

Recently, CDX2 reduction has been related to the aggressive phenotype of tumor cells, including proliferation, apoptosis, invasion, migration, drug resistance^40^, cell stemness^41^, and differentiation^42^. An unlimited proliferation capability is the hallmark of tumor cells^43^. The roles of CDX2 in cell proliferation were reported in multiple cancers. Down-regulation of CDX2 expression promoted gastric cancer cell proliferation^44^. In lung cancer cells, restored CDX2 expression suppressed cell proliferation by arresting the cell G1/S transition^24^. Our previous study revealed that in colon cancer cells, overexpression of CDX2 significantly inhibited cell viability and xenograft tumor formation^30,31^. However, Zhu et al. reported that CDX2 promoted HCC cell proliferation by directly transactivating the oncogene CDH-17^45^. In gastric cardia cancer tissues, high expression of CDX2 is positively correlated with cell proliferation marker Ki67^46^. These findings highlight the need to dissect and characterize the precise roles of CDX2 in different cancer types.

The precise molecular mechanism involved in the CDX2-mediated inhibition of colon cancer cell proliferation was investigated in our study. Our study showed that CDX2 knockdown promoted cell proliferation in vitro, accelerated tumor formation in vivo, and induced the G1/S cell cycle transition, whereas overexpression of CDX2 suppressed cell proliferation, suggesting that CDX2 inhibits proliferation in colon cancer cells.

The evolutionarily conserved Wnt signaling performs a crucial role in embryonic development and tissue homeostasis^8^. In addition, mounting evidence indicates that activated Wnt signaling is implicated in the tumourigenesis of various human cancers^47–49^. The association between Wnt signaling and intestinal disorders has been recognized in CRC and inflammatory bowel diseases (IBD). Coskun reported that in colon cancer cells, CDX2 suppressed the Wnt signaling activity^50^. However, whether CDX2 inhibits the proliferation and tumor formation of colon cancer cells by suppressing the activity of the Wnt signaling was not fully addressed. In the present study, the TOP/FOP-Flash reporter assay showed that CDX2 knockdown or CDX2 overexpression led to enhanced or attenuated Wnt signaling activity in colon cancer cells. Furthermore, for the genes downstream of Wnt signaling, the levels of the corresponding proteins, including β-catenin, cyclin D1, and c-myc, were up-regulated or down-regulated in CDX2-knockdown or CDX2-overexpressing cells. The CDX2-mediated inhibition of Wnt/β-catenin signaling and the subsequent cellular proliferation have been reported in lung cancer^24,51^. Our study reveals that CDX2 inhibits the proliferation of colon cancer cells by suppressing Wnt signaling activity. In different cancer types, the molecular mechanism by which CDX2 regulates tumor cell proliferation is intricate. In pancreatic cancer cells, CDX2 inhibits cell proliferation by directly repressing cyclin D1 transcriptional activity^22^. Bai et al.^26^ reported that CDX2 might regulate PTEN, an important tumor suppressor, thereby inhibiting the gastric cancer aggressive biological phenotype via PI3K/Akt pathway.

A genome-wide CDX2 ChIP-Seq analysis of Caco-2 cells revealed that CDX2 binds to an upstream enhancer of APC and AXIN2 and the promoter of GSK-3β^34^. Our study found CDX2 knockdown or CDX2 overexpression led to a decrease or increase in GSK-3β and Axin2 expression. However, no significant change in APC expression was observed in CDX2-modified cells, which is inconsistent with the results of Olsen et al.^38^. The dual-luciferase reporter assay confirmed that CDX2 transactivated the promoter activity of GSK-3β and Axin2. Furthermore, our study demonstrated that CDX2 specifically and directly binds to the GSK-3β promoter and the Axin2 upstream enhancer in colon cancer cells, by qChIP. Olsen et al.^38^ reported that CDX2 did not affect the GSK-3β expression in Caco-2 cells, although CDX2 binds to the GSK-3β promoter. However, down-regulation of CDX2 following TNF-α treatment suppressed GSK-3β expression^52^. The inconsistent results might be related to the fact that other transcription factors also regulate GSK-3β expression.

In addition to the transcriptional regulation mechanism of CDX2 regulating Wnt signaling mentioned above, Guo et al. found that CDX2 could interact with β-catenin and disturb the transcriptional ability of β-catenin/TCF^53^. CDX2 regulated the protocadherin Mucdhl, a β-catenin-interacting protein, and subsequently prevented the nuclear translocation of β-catenin^54^. These studies indicated that the molecular mechanism of CDX2 suppressing Wnt signaling is intricate.

In addition, suppression of Wnt signaling by XAV-939 abolished the growth and viability of colon cancer cells induced by CDX2 knockdown. Conversely, treatment with CHIR-99021 led to a marked recovery in cell growth and viability suppressed by CDX2 overexpression. These data support the idea that CDX2 inhibits the proliferation and tumor formation of colon cancer cells by suppressing Wnt signaling. The result of IHC staining in human CRC specimens showed that CDX2 expression was inversely associated with β-catenin, cyclin D1, and c-myc expression, further supporting the notion that CDX2 is a negative regulator of Wnt signaling.

In summary, our study demonstrates that CDX2 inhibits the proliferation and tumor formation of colon cancer cells by suppressing Wnt signaling activity and reveals a molecular mechanism by which CDX2 regulates the transcriptional activation of GSK-3β and Axin2.

## Supplementary information

Supplementary Figure Legend

Figure S1

Figure S2

Figure S3

Figure S4

Figure S5
