# Supplementary material for: CDX2 inhibits the proliferation and tumor formation of colon cancer cells by suppressing Wnt/β-catenin signaling via transactivation of GSK-3β and Axin2 expression
Source: Cell Death Dis. 2019 Jan 10;10(1):26. doi: 10.1038/s41419-018-1263-9 (PMC6328578; doi:10.1038/s41419-018-1263-9)
Supplement: Supplementary file 1 — Supplementary Figure Legend [file 41419_2018_1263_MOESM1_ESM.docx]

Supplementary Fig. 1 The effects of up-regulation and down-regulation of CDX2 expression in colon cancer cells. a, b. Western blot bands showing the CDX2 expression in CDX2-knockdown and CDX2-overexpressing HT-29 (a) and Caco-2 (b) cells. c, d. Quantitative analysis of CDX2 expression in CDX2-knockdown and CDX2-overexpressing HT-29 (c) and Caco-2 (d) cells. All data are the mean±SD of three independent experiments. * P<0.05.

Supplementary Fig. 2 CDX2 attenuates the activity of the Wnt/β-catenin pathway in colorectal carcinogenesis. a, b. Quantitative analysis of the cyclin D1, c-myc and β-catenin protein expression in CDX2-knockdown (a) and CDX2-overexpressing (b) HT-29 cells. c, d. Quantitative analysis of the cyclin D1, c-myc and β-catenin protein expression in CDX2-knockdown (c) and CDX2-overexpressing (d) Caco-2 cells. All data are the mean±SD of three independent experiments. * P<0.05.

Supplementary Fig. 3 Blockage of the Wnt/β-catenin pathway by XAV-939 attenuates the cell proliferation promoted by CDX2 knockdown. a, b. Western blot bands of cyclin D1, c-myc and β-catenin in CDX2-knockdown HT-29 (a) and Caco-2 (b) cells treated with a β-catenin inhibitor, XAV-939. c, d. Quantitative analysis of the cyclin D1, c-myc and β-catenin protein expression in CDX2-knockdown HT-29 (c) and Caco-2 (d) cells treated with XAV-939. e, f, g, h. Cell growth and MTT assays evaluating the proliferation and viability of CDX2-knockdown HT-29 (e, f) and Caco-2 (g, h) cells treated with XAV-939. All data are the mean±SD of three independent experiments. * P<0.05.

Supplementary Fig. 4 Activation of the Wnt/β-catenin pathway by CHIR-99021 enhanced the cell proliferation suppressed by CDX2 overexpression. a, b. Western blot bands of cyclin D1, c-myc, β-catenin and GSK-3β in CDX2-overexpressing HT-29 (a) and Caco-2 (b) cells treated with CHIR-99021, a GSK-3β inhibitor. c, d. Quantitative analysis of cyclin D1, c-myc, β-catenin and GSK-3β in CDX2-overexpressing HT-29 (c) and Caco-2 (d) cells treated with CHIR-99021.e, f, g, h. Cell growth and MTT assays evaluating the proliferation and viability of CDX2-overexpressing HT-29 (e, f) and Caco-2 (g, h) cells treated with CHIR-99021. All data are the mean±SD of three independent experiments. * P<0.05.

Supplementary Fig. 5 CDX2 regulates the expression of GSK-3β and Axin2 in colon cancer cells. a, b. Quantitative analysis of the APC, Axin2 and GSK-3β protein expression in CDX2-knockdown (a) and CDX2-overexpressing (b) HT-29 cells. c, d. Quantitative analysis of the APC, Axin2 and GSK-3β protein expression in CDX2-knockdown (c) and CDX2-overexpressing (d) Caco-2 cells.
