# Supplementary figures and images for: CDX2 inhibits the proliferation and tumor formation of colon cancer cells by suppressing Wnt/β-catenin signaling via transactivation of GSK-3β and Axin2 expression
Source: Cell Death Dis. 2019 Jan 10;10(1):26. doi: 10.1038/s41419-018-1263-9 (PMC6328578; doi:10.1038/s41419-018-1263-9)

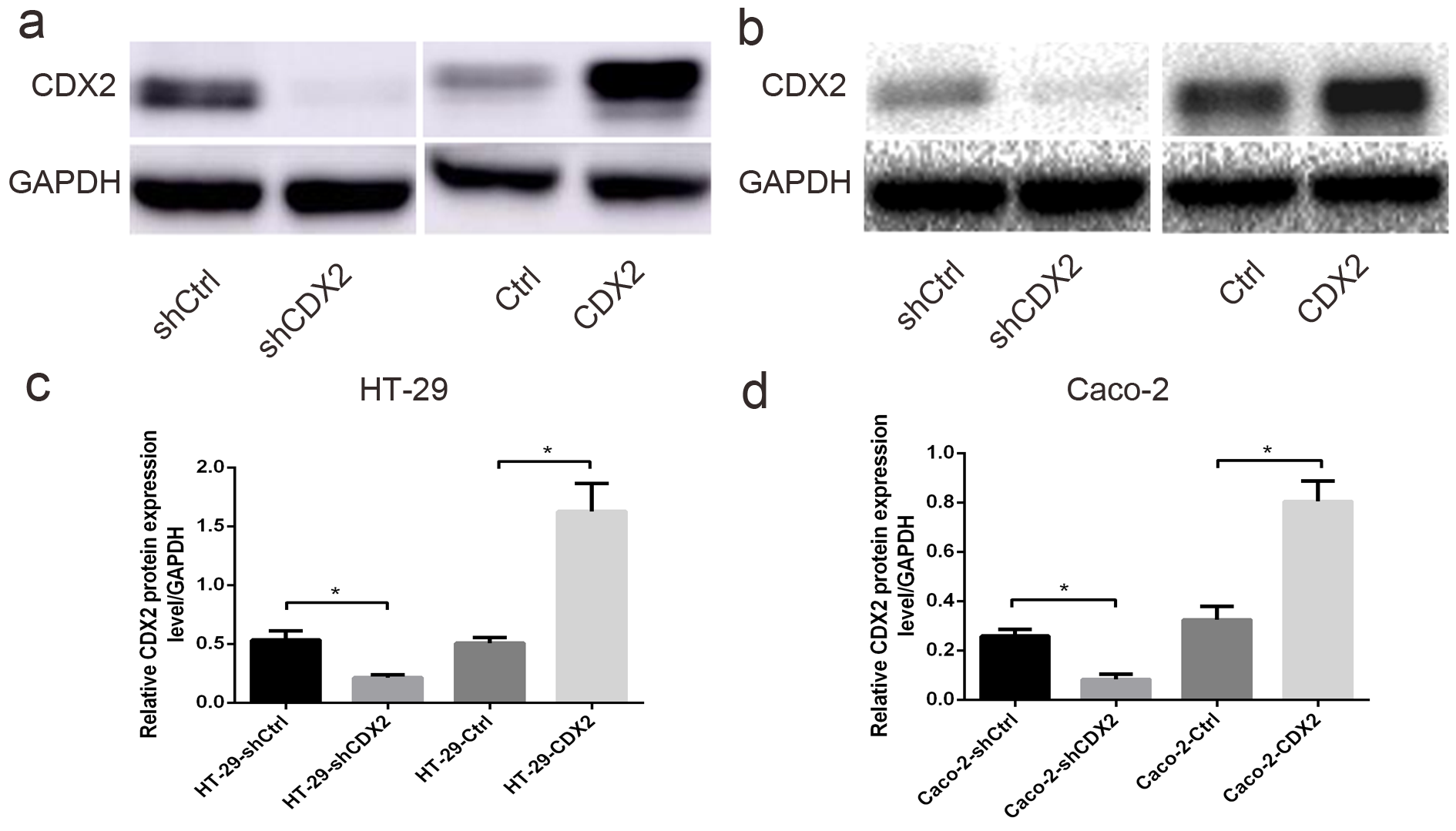

Supplement: Supplementary file 2 — Figure S1 [file 41419_2018_1263_MOESM2_ESM.tif]

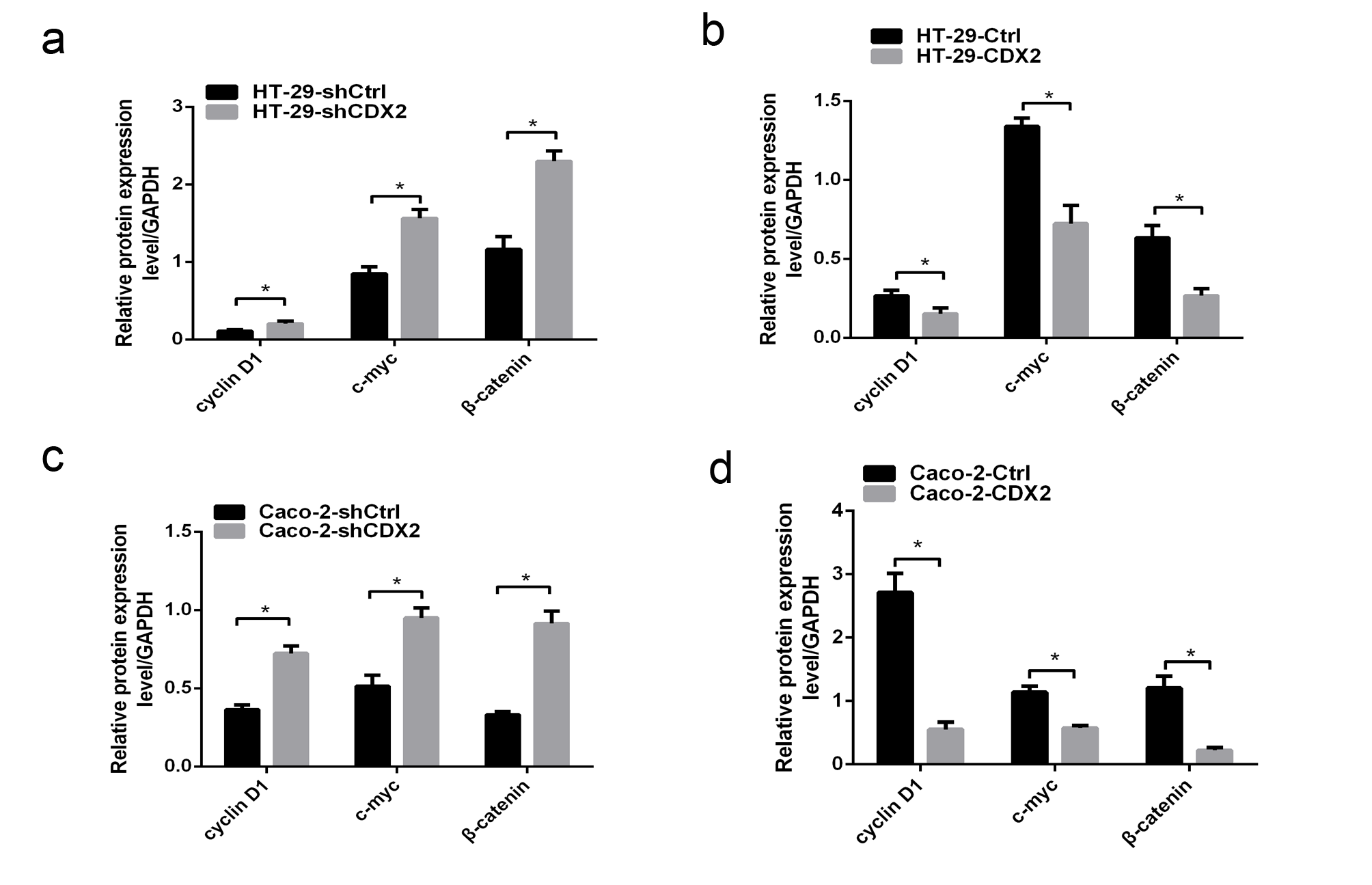

Supplement: Supplementary file 3 — Figure S2 [file 41419_2018_1263_MOESM3_ESM.tif]

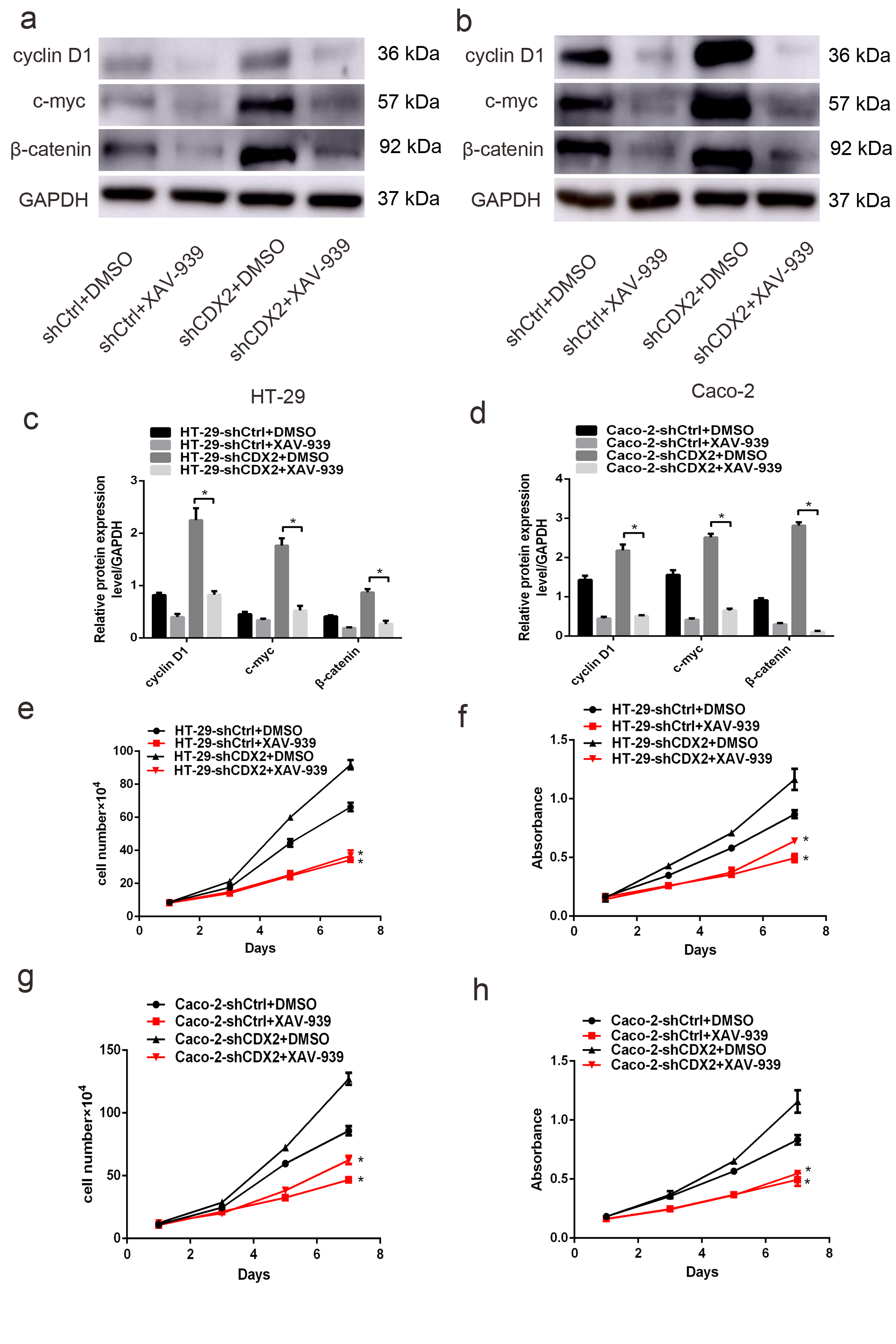

Supplement: Supplementary file 4 — Figure S3 [file 41419_2018_1263_MOESM4_ESM.tif]

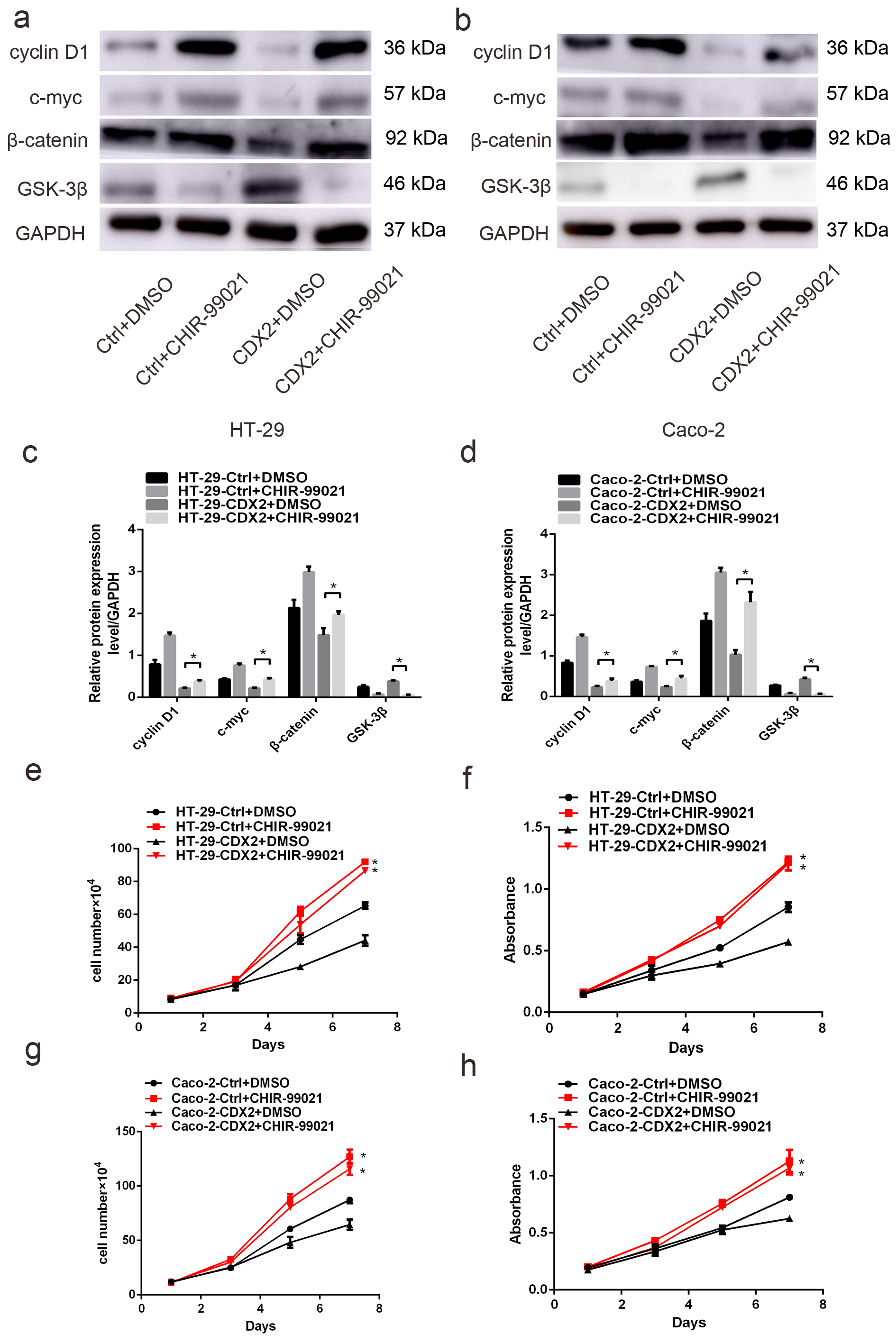

Supplement: Supplementary file 5 — Figure S4 [file 41419_2018_1263_MOESM5_ESM.tif]

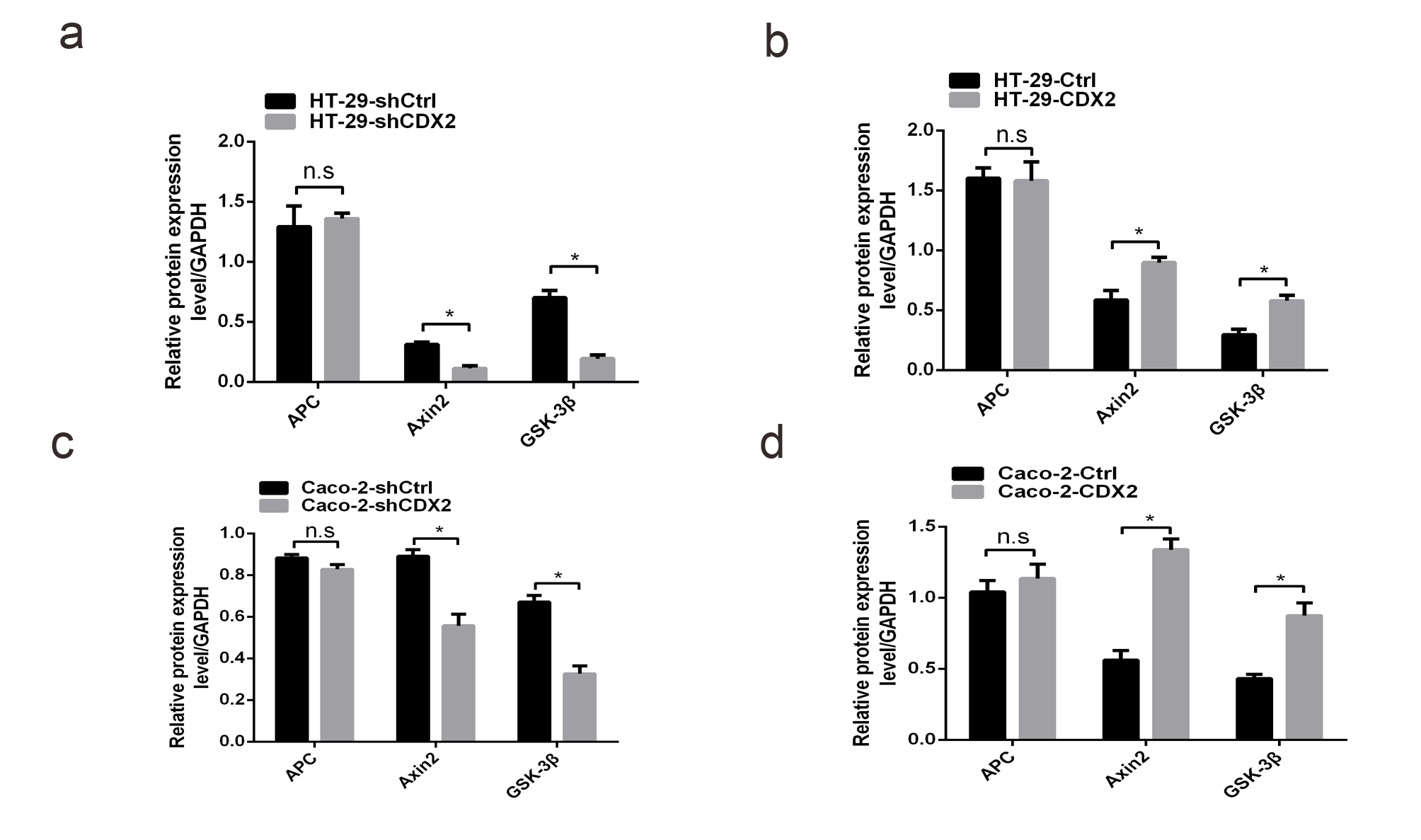

Supplement: Supplementary file 6 — Figure S5 [file 41419_2018_1263_MOESM6_ESM.tif]
